# The role of sand in an impact-based bedload monitoring system

**DOI:** 10.1038/s41598-026-53231-x

**Published:** 2026-05-26

**Authors:** Manuel Pirker, Hannes Badura, Josef Schneider, Stefan Haun

**Affiliations:** 1 Institute of Hydraulic Engineering and Water Resources Management, Graz University of Technology, Stremayrgasse 10/II, 8010 Graz, Austria; 2 VERBUND Hydro Power GmbH, Europaplatz 2, 1150 Vienna, Austria

**Keywords:** Bedload monitoring, Sediment transport, Acoustic impact sensor, Environmental sciences, Hydrology, Natural hazards, Solid Earth sciences

## Abstract

Monitoring bedload transport is essential for understanding the morphology of rivers, yet traditional direct measurement techniques offer limited spatial and temporal resolution. Impact plates are a valuable addition for monitoring the transport of gravel-sized particles, although they require extensive field calibration. Recent research has therefore focused on developing globally applicable calibration approaches derived from controlled laboratory experiments. These efforts mainly aim to reproduce field-like flow velocities and roughness conditions. One aspect that remains largely unexamined is the influence of coincident sand transport. To address this gap, we conducted laboratory flume experiments that included additional sand transport, thereby replicating natural conditions during flood events more closely. Three sand feeding rates and seven gravel-sized diameter classes were investigated under high flow velocities, as they occur in mountainous rivers and creeks. The results were then compared with clear-water experiments. Signal and video analyses revealed that frequency-based signal properties, particularly centroid frequency, are strongly affected by the transported sand, whereas amplitude-based properties are more robust. Moreover, sand introduces biases in transport rate predictions derived from impulse counts. This study highlights the need to account for sand transport when calibrating impact-based monitoring systems, ultimately supporting more effective riverine sediment management and future revitalization measures.

## Introduction

The disruption of sediment continuity in rivers poses wide-ranging consequences for life in the river and on the surrounding lands. Socioeconomic challenges such as flood safety^1,2^ and delta degradation^3,4^, combined with ecological factors^5,6^, have driven the ongoing development and improvement of monitoring techniques for bedload fluxes.

Traditionally, direct monitoring techniques such as mobile bed samplers and bottom traps were used. These methods can provide information both on sediment flux and on grain size distribution, though they provide only punctual measurements. To provide broader insights into sediment transport processes, they can be deployed in combination with indirect measurement techniques like ADCP bottom track^7,8^, dune tracking with echo sounders^9,10^, as well as seismic^11–13^ and hydro-acoustic methods^14–17^.

Although these indirect measurement systems enable continuous measurements, they come with their own limitations. Similar to basket samplers, ADCPs and echo sounders cannot be deployed under high-flow conditions. Additionally, like seismic methods, they do not provide information on the grain size distribution of the transported sediments. To date, hydro-acoustic methods are the only indirect methods that are able to infer this kind of information while also remaining operable independent of flow conditions. Hydro-acoustic methods infer statistical mass transport and grain diameters either by the self-generated noise of colliding particles^16,17^ or by the vibrations caused when particles impact on a pipe^18–21^ or a plate^22^.

Impact plate systems consist of an acoustic sensor, commonly an accelerometer^23,24^, a microphone^25,26^, or a geophone^14,27,28^ mounted underneath a steel plate, which records noise and oscillations caused by impacting particles on the plate. In detail, accelerometers measure resistive or capacitive changes resulting from the deformation of a small sensing structure, which are, in turn, proportional to the applied acceleration. They offer a broad frequency response and are sensitive to high-frequency components, making them potentially more suitable for detecting smaller particles^29^. Similarly, microphones convert sound pressure waves into electrical signals through the deformation of a flexural disc, responding to pressure variations rather than acceleration. In contrast, geophones measure a velocity-proportional current induced by the relative movement of a magnet in a coil, making them less sensitive to high-frequency vibrations.

These impact-based systems have been widely deployed to obtain continuous bedload information across diverse applications. For example, recent studies have deployed Japanese Pipe Microphones (JPM) to evaluate the performance of movable shutter to control sediment runoff during floods^21,30^. Furthermore, Swiss Plate Geophones (SPG) are used to investigate the temporal and spatial variability of bedload transport over the past decades in mountain streams^31^, as well as Alpine^32,33^ and gravel-bed rivers^34,35^. Also, Lammer et al. (2025)^36^ evaluated bedload transport equations on data collected in an Alpine river, acknowledging the importance of long-term continuous measurements to improve existing calculation approaches. Further applications include the deployment of impact plates to monitor bedload transport through sediment bypass tunnels^37,38^.

SPGs in particular have been extensively studied under both laboratory and field conditions, with substantial advancements made towards a general calibration solely derived from laboratory experiments^28^. Particular focus was placed on the application of signal processing techniques. Barrière et al. (2015)^26^ derived grain size information from impact signal characteristics, namely amplitude and centroid frequency. Wyss et al. (2016)^39^ continued this work and analyzed the recorded signals in terms of signal packets, which represent one complete oscillation associated with a particle impact, rather than in terms of single impulses. They were able to infer the diameter of the passing particles based on statistical packet properties. Furthermore, Nicollier et al. (2022)^40^ used packet properties to create a filter technique to reduce ambient noise caused by apparent impacts, i.e., particles impacting beside the sensor plate, and used it to derive a general calibration for the SPG system across multiple streams. Additionally, Chen et al. (2022)^41^ investigated the structural response of the impact-plate system itself, using finite-element modeling combined with flume experiments, and inferred sediment transport modes from registered packet properties.

All these studies investigated grains in natural and spherical shapes, with diameters ranging from 10 to 150 mm. They included both data derived from laboratory flumes and actual field measurements. While field studies included both clear-water calibrations and sand-laden flows during events, the laboratory investigations have primarily focused on replicating field-like flow conditions based on flow velocity, bed roughness, and, to a lesser extent, water depth. The influence of sand, which is commonly present under natural flow conditions, has not yet been examined under controlled conditions.

To address this gap, this study investigated impact plate accelerometers (IPA) to determine whether the presence of sand affects (i) the capability of particle diameter estimation from signal properties, and (ii) the statistical mass transport estimates of the gravel-sized particles. Both represent key factors to quantify bedload transport, making them primary targets for estimations based on indirect bedload monitoring systems.

## Methods

We conducted laboratory experiments to assess the influence of sand on the predictive capabilities of IPAs under high flow conditions. Following the calibration procedure described by Wyss et al. (2016)^27^ and Nicollier et al. (2022)^40^, five flow velocities of 2.7 to 5.1 $\text{m s}^{-1}$ were tested in clear water conditions, using seven gravel particle classes with diameters between approximately 15 and 60 mm. Gravel particles were introduced into the flume at feeding rates of 0.02 and 0.71 $\text{kg s}^{-1}$, and the resulting impulses were recorded by the IPA.

This procedure was then repeated under sand-dominated transport conditions, with coarse sand introduced at three feeding rates between 0.90 and 1.80 $\text{kg s}^{-1}$.

In total, each velocity setting was repeated ten times, yielding 50 runs of five seconds duration for each particle-diameter class and sand feeding rate. Overall, 1,400 runs were conducted across the four experiments.

### Experimental setup

**Fig. 1:**
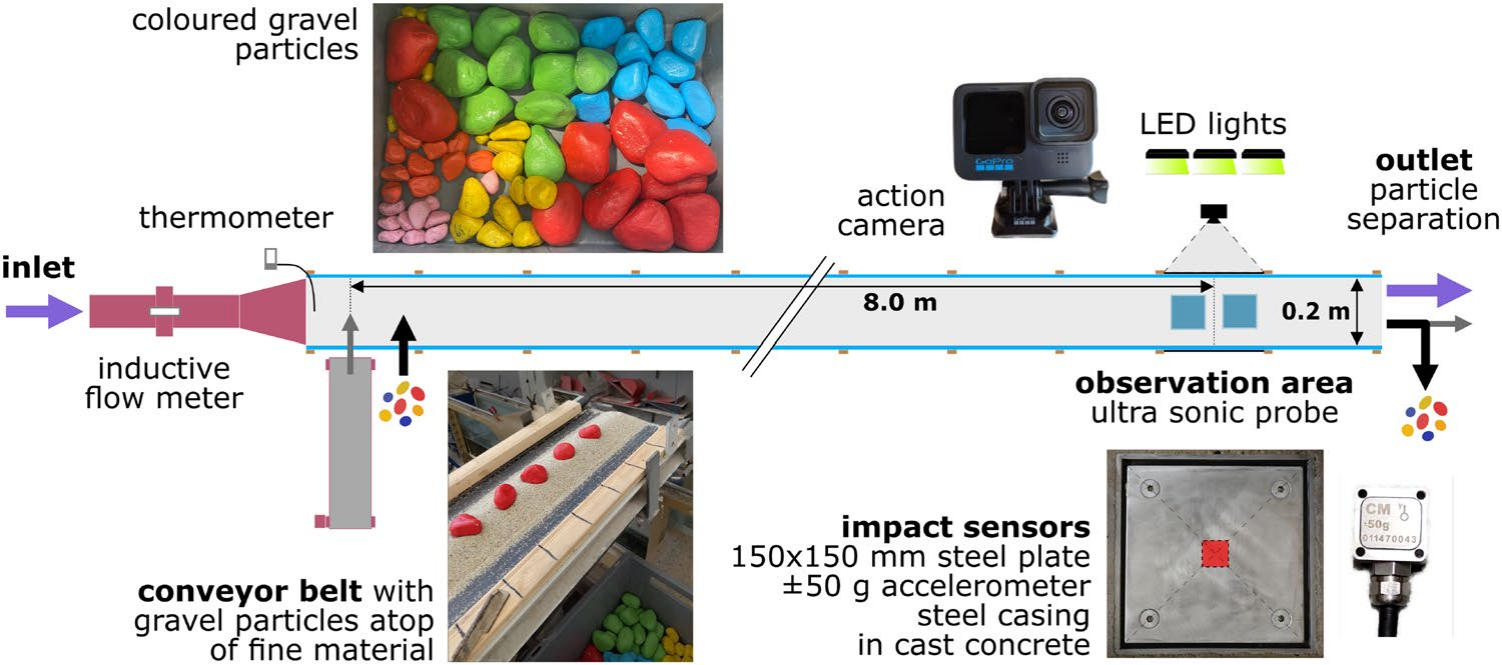
Plan view of the experimental flume setup showing the locations of the measurement devices, the conveyor belt for feeding sand, and the observation area with two impact sensors.

A schematic overview of the experimental setup is shown in Fig. 1. The experiments were conducted in a 12 meter long and 0.2 meter wide flume with glass walls and a smooth concrete bed inclined at 1 %. The roughness of the concrete bed was determined by repeated water level measurements along the flume during multiple stationary flow conditions and fitting of a backwater curve. This fitting resulted in a sand-grain roughness $k_s$ of 0.15 to 0.20 mm, falling within the range reported in the literature for similar setups^42^.

Two IPAs were installed in series at the outlet of the flume, plane with its bed. Each of these consisted of a uniaxial IAC-CM-I-01 accelerometer from Micromega’s Recovib series that recorded vibrations caused by impacting particles. These capacitive accelerometers are based on a micro-electromechanical system (MEMS) in which the deformation of a flexible structure changes the measurable capacitance, which is proportional to the applied acceleration.

Both sensors operated at a sampling frequency of 10 kHz and a measurement range of *± 50* g, approximately *± 500* $\text {m s}^{-2}$. Each accelerometer was mounted at the underside of a 150 by 150 mm stainless steel plate with a thickness of 5 mm. The steel plate was fixed to a steel casing with four screws, separated by a 15 mm thick polymer layer to isolate it from ambient vibrations. The steel casing itself was cast into a 40 kg concrete block, which was connected to the flume’s concrete bed.

Figure 1 also displays the location of the measurement devices used to calculate flow conditions. Discharge was continuously monitored at the flume inlet using an ABB COPA-XE DE43F inductive flowmeter. Water temperature was measured manually using a Mastech MS6501 thermometer. A Microsonic mic25+ ultrasonic distance sensor was installed to measure water depth in the observation area near the impact sensors. The measured values were averaged over each five-second run, and the corresponding flow properties were subsequently calculated. Table 1 summarizes the mean flow conditions based on these measurements for all four experiments. The pressurized flume inlet led to a supercritical, decelerating flow, with an averaged discharge over the run time *Q* ranging from 0.075 to 0.155 $\text {m}^3\text {s}^{-1}$, leading to mean velocities *U* above the impact sensors of 2.68 to 5.11 $\text {m s}^{-1}$. The corresponding water depth above the sensors $h_w$ was between 0.142 and 0.151 $\text {m}$. The shear velocity $U^*$ ranged from 0.114 to 0.211 $\text {m s}^{-1}$. Furthermore, the roughness Reynolds number $k_s^+$, representing the ratio of roughness height of the flume bed to the viscous sublayer thickness, was calculated to classify the flow regime. All experiments fall within the transitionally rough regime, with $k_s^+$ ranging from 20 to 33 according to Nikuradse (1932)^43^.

Further details on how these hydraulic parameters were determined are available in the supplementary material in the section *Hydraulic parameters*.

**Table 1:** Averaged hydraulic flow parameters for each tested velocity class and mass transport capacity of the fed sand $\hat{Q}_{s}$. Percentage values (%) represent the relative standard deviation over all experiments, indicating variability.

Velocity class	Measured discharge	Measured water level	Flow velocity	Froude number	Reynolds number	Shear velocity	Roughness Reynolds	Transport capacity
	*Q* (m$^3$s$^{-1}$)	$h_w$ (m)	*U* (m s$^{-1}$)	Fr	Re	$U^*$ (m s$^{-1}$)	$k_s^+$	$\hat{Q}_{s}$ (kg s$^{-1}$)
1	0.076 ± 0.8*%*	0.142 ± 2.8*%*	2.68	2.27	6.38e+05	0.114	19.6	19.2
2	0.096 ± 0.5*%*	0.147 ± 1.1*%*	3.26	2.72	7.76e+05	0.137	23.3	33.4
3	0.116 ± 0.5*%*	0.146 ± 3.3*%*	3.95	3.30	9.07e+05	0.165	27.1	61.0
4	0.135 ± 0.5*%*	0.147 ± 4.3*%*	4.60	3.84	1.04e+06	0.191	30.9	97.7
5	0.153 ± 0.6*%*	0.151 ± 3.4*%*	5.06	4.16	1.12e+06	0.208	32.6	124.9

Seven particle classes were defined based on their mean diameter $D_j$ and mass $m_{g,j}$, ranging from 13.9 to 66.5 mm and 4.7 to 260 grams per particle, respectively. Summary statistics of each class are provided in Table 2. To define these classes, particles were preselected by a contrast-based image sieving technique. This technique was implemented using the Python library OpenCV. First, the contours of the gravel particles were identified in rectified and scaled images. Then an ellipse was fitted to each contour, where the extracted major and minor axes represent the particles’ A- and B-axes, respectively. Particles were selected if (i) their B-axis diameter deviated no more than 20 % from the class mean, and (ii) their axes ratio exceeded 0.5, to exclude overly elongated particles.

**Table 2:** Gravel particle size classes.

Particle class	Particle diameter $D_{j}$ (mm)		A/B-axis ratio (-)		Particle mass $m_{g,j}$ ($10^{-3}$ kg)		mass of gravel particles per run	Gravel feeding rate
*j*	min - max	mean	min - max	mean	min - max	mean	$M_{g,j}$ (kg)	$Q_{g,j}$ (kg s$^{-1}$)
1	13.9 - 18.3	16.5	0.50 - 0.96	0.78	4.7 - 6.1	5.4	0.08	0.016
2	18.3 - 22.2	20.2	0.53 - 0.99	0.72	9.7 - 13.1	11.4	0.17	0.034
3	21.3 - 26.1	24.2	0.62 - 0.94	0.74	17.1 - 21.3	18.9	0.28	0.057
4	27.4 - 36.6	31.9	0.58 - 0.90	0.75	37.0 - 44.0	40.6	0.61	0.122
5	37.4 - 47.2	41.2	0.63 - 0.95	0.81	76.0 - 88.0	80.8	1.21	0.242
6	41.6 - 58.6	51.2	0.62 - 0.96	0.80	145.0 - 172.0	157.8	2.37	0.473
7	50.1 - 66.5	56.9	0.57 - 0.96	0.77	215.0 - 260.0	235.9	3.54	0.708

Sedimentological characteristics of the investigated size classes, including the total gravel mass passing each sensor per run $M_{g,j}$ and the corresponding mass transport rates $Q_{g,j}$ for a run duration of 5 seconds.

A GoPro Hero 8 action camera was mounted facing the flume, perpendicular to the side wall, and centered between the impact sensors in the observation area (see Fig. 1). It recorded the trajectories of passing gravel particles, which were colored according to their class. This coloring helped to increase the contrast of the backside of the flume as well as the transported sand. Additional information on the camera setup and the video processing follows in Chapter *Video processing*.

A conveyor belt was installed 8 meters upstream of the impact sensors, near the flume inlet. It was used to feed coarse sand with grain diameters of 1 to 2 mm and a bulk density of 1600 $\text{kg m}^{-3}$ into the flume. The feeding rate was calibrated by adjusting the conveyor belt velocity, while sand was delivered onto the belt through a fixed opening. The mass of sand was discharged over a fixed five-second interval for multiple constant belt velocities. The mass of the discharged sand was then measured to establish a relationship between belt velocity and mass feeding rate.

Gravel particles were manually placed on top of the sand at equal intervals (see Fig. 1). The conveyor belt introduced the gravel particles in the flume at a feeding rate $Q_g$ proportional to particle diameter, ranging from 0.02 to 0.71 $\text{kg s}^{-1}$, corresponding to a unit gravel mass transport rate $q_g$ of 0.08 to 3.54 kg m$^{-1}$s$^{-1}$. This arrangement ensured that when particles were partially or completely obscured by the sand during multiple frames, their trajectory could still be traced. A limitation of this approach was that the number of particles per class had to be fixed at 15 to keep the required amount of sand manageable. Despite this restriction, 6.6 tons of sand were required for the experiments.

A feeding time of five seconds was determined for each class of 15 particles during preliminary experiments. This duration included a buffer to ensure continuous sand transport while the particles passed the sensor location near the flume outlet.

Four experiments were conducted in total, i.e. one in clear water conditions as reference and three with different sand feeding rates $Q_s$ of 0.90, 1.35, and 1.80  kg s$^{-1}$, corresponding to unit sediment transport rates $q_s$ of 4.50, 6.75, and 9.00 kg m$^{-1}$s$^{-1}$, respectively. This investigated range lies between 0.7 and 9.9 % of the mass transport capacity for according to the corrected Meyer-Peter-Müller formula^44^, which ranges from 19 to 125 kg s$^{-1}$ (see Table 1). Each experiment included five runs conducted at different flow velocities. All seven particle classes were tested during each run and collected in a mesh box at the outlet of the flume, separated from the transported sand. Moreover, each run was repeated 10 times, resulting in a cumulative gravel particle mass passing over a single impact sensor as listed in Table 2. Therefore, 50 runs were performed for each sand feeding rate and particle diameter class.

### Signal processing

Sensor signals were recorded using National Instruments hardware and their official Python API. Signal processing was performed in Python using the scipy.signal library. A Butterworth high-pass filter with a cutoff frequency of 75 Hz was applied to suppress low-frequency vibrations. Signals were initially sampled at 30 kHz, but frequency components above the accelerometer’s valid range (10 kHz) were subsequently filtered out to avoid aliasing and noise.

#### Impulses

Following Rickenmann and McArdell (2007)^14^, an impulse is defined as an instance when the recorded signal exceeds a predefined threshold. A threshold of 1 g (9.81 m s$^{-2}$) was selected because this value represents 1 % of the sensor measurement range and is one order of magnitude greater than the standard deviation of the background noise associated with turbulent flow. For all tested flow velocities and sand feeding rates, background noise remained below 0.05 g ( 0.50 m s$^{-2}$).

#### Packets

The processing of the accelerometer signals is based on the procedure developed by Wyss et al. (2016)^39^ for geophone signals. This procedure has been widely applied to improve understanding of impact-based bedload measurement principles^27,28,45,46^. In summary, a Hilbert transform combined with a threshold is used to detect signal packets caused by impacting particles onto the sensor plate. Only packets with a duration longer than 1 millisecond are processed. The packet edges are smoothed using a Tukey window to avoid the introduction of artificial frequency components. Then, the packets are zero-padded to a uniform length of 100 milliseconds. Analogous to impulse detection, the threshold to detect these signal packets was also chosen with 1 g.

### Diameter estimation

Particle diameters were estimated from signal packet properties using a power-law relationship. In its general form, this relationship is defined as:

1 $$\begin{aligned} D_{j, est} = a\cdot \text {agg}(X_j)^b \end{aligned}$$

Where $D_{j, est}$ is the estimated diameter, *a* and *b* are regression coefficients, $\text {agg}(X_j)$ is the aggregate of the regressor variable over all runs conducted with diameter class *j*. In this work, the median of the packet properties is used as the aggregation function.

The regressor variables were selected from signal characteristics commonly employed for particle-size estimation in impact-plate systems. These include the centroid frequency $f_{centroid}$, amplitude-based descriptors derived from the signal envelope, and packet length. The selection follows previous studies on impact sensors^27,40,45,46^.

#### Centroid frequency

The centroid frequency $f_{centroid}$ is the average frequency weighted by the Fourier amplitudes and represents the center of mass of the Fourier spectrum. It is calculated by:

2 $$\begin{aligned} f_{centroid}=\frac{\sum _i f_i\cdot A_i}{\sum _i A_i} \end{aligned}$$

Where $f_i$ and $A_i$ are the Fourier frequencies and amplitudes, respectively.

#### Envelope amplitude properties

Further packet properties were derived from the Hilbert envelope of the packet’s acceleration signal. From this envelope, the maximum amplitude $A^{max}_{env,acc}$ was extracted, together with selected percentiles of the envelope amplitude distribution, namely $A^{95th}_{env,acc}$, $A^{85th}_{env,acc}$, $A^{75th}_{env,acc}$, $A^{50th}_{env,acc}$. Additionally, the original signal was integrated to retrieve corresponding velocity and displacement signals. The Hilbert envelopes of these derived signals were computed, yielding $A^{max}_{env,vel}$ and $A^{max}_{env,dis}$, as well as their respective percentiles.

### Bedload discharge estimation

Bedload transport rates were estimated independently using impulse counts and packet counts.

The impulse-based bedload transport rate $Q^{imp}_{b,est}$ was calculated as

3 $$\begin{aligned} Q^{imp}_{b,est} = \frac{N^{imp}}{k^{imp}_{b}\cdot \Delta t} \end{aligned}$$

Where $N^{imp}$ denotes the total number of registered impulses during the observation interval *Δ t*, and $k^{imp}_{b}$ is the impulse-per-mass coefficient.

The coefficient $k^{imp}_{b}$ was determined by fitting a linear relationship between the number of registered impulses per experimental run for particle size class *j*, $N^{imp}_{j}$, and the corresponding total transported gravel mass $M_{g,j}$:

4 $$\begin{aligned} N^{imp}_{j} = k^{imp}_{b}\cdot M_{g,j} \end{aligned}$$

Analogously, the packet-based bedload transport rate $Q^{pack}_{b,est}$ was computed as

5 $$\begin{aligned} Q^{pack}_{b,est} = \frac{N^{pack}}{k^{pack}_{b}\cdot \Delta t} \end{aligned}$$

Where $N^{pack}$ is the total number of segmented signal packets during *Δ t*, and $k^{pack}_{b}$ denotes the packet-per-mass coefficient. It was obtained from the linear relationship between the number of registered packets per run of particle size class *j*, $N^{pack}_{j}$, and the corresponding transported gravel mass $M_{g,j}$:

6 $$\begin{aligned} N^{pack}_{j} = k^{pack}_{b}\cdot M_{g,j} \end{aligned}$$

### Video processing

The action camera recorded the experimental runs with a frame rate of 240 frames per second and an exposure time of 1 ms. To minimize caustic effects and ensure consistent lighting, the top of the flume was covered during recording, and three LED flood lights were positioned laterally to illuminate the observation area. The camera was placed at a distance of 50 cm from the flume, causing a notable distortion effect. This distortion was removed during pre-processing using reference markers. Synchronization between video clips and measurement signals was achieved using a single LED that remained lit throughout each experimental run. The video processing, object segmentation, and trajectory tracking were implemented in Python by using the OpenCV library.

Bedload consists of particles in rolling, sliding, or saltation motion^47^. These transport modes were identified for the individual gravel particle based on the trajectory of its contour centroids across consecutive video frames. To extract this trajectory, a sequence of 24 frames was used, centered at the moment of impact identified in the accelerometer signal. First, the particles are classified as being in saltation or in a non-saltation mode, i.e., rolling and sliding, which are not explicitly distinguished in this analysis. A particle was considered as being in saltation if the vertical contour centroid, *y*, exceeded a height of $0.6\cdot D_m$ above the flume bed^48^, where $D_m$ corresponds to the mean diameter of the investigated gravel particle diameter class. A graphical depiction of this process is provided in Supplementary Fig. S1.

## Results

### Video analysis

Table 3 shows the transport modes identified per signal packet via video analysis, as well as the share of packets caused by particles in saltation, which directly impacted the sensor. $N_{total}$ represents the total number of signal packets caused by either direct or apparent impacts of gravel particles on the two sensor plates. This number decreased by 16.4 % compared to clear water conditions when sand was introduced with the lowest feeding rate of 0.90 kg s$^{-1}$. Closer examination of the recorded signal packets revealed that a number of them were composed of overlapping oscillations generated by successive particle impacts. The original envelope-based detection method was unable to resolve these individual impacts. Therefore, any packet displaying a secondary peak was automatically split, using the prominence of the peaks as the criterion. This correction increased the total number of packets ($N_{total}^{corr}$) by 2.5 % in clear water conditions, but by 18.5, 23.4, and 24.9 %, for feeding rates of 0.90, 1.35, and 1.80 kg s$^{-1}$, respectively. Furthermore, while the number of particles over all diameter classes identified in saltation $N_{salt}$ remained relatively stable between 2714 and 3142, the number of rolling or sliding particles $N_{slide,roll}$ continuously decreased with an increasing sand feeding rate from 2133 to 1355. This decrease is likely due to the reduced detection rate of particles traveling close to the bed, where most of the sand is concentrated.

**Table 3:** Summary of identified gravel particle motions.

Sand feeding rate	Total number of packets		Packets associated with particle motion			Packets associated with direct impact on sensor
			Unclassified	Sliding or rolling	Saltation	
$Q_s$ (kg s$^{-1}$)	$N_{total}$	$N_{total}^{corr}$	$N_{uncl}$	$N_{slide,roll}$	$N_{salt}$	$N_{salt,direct}$
0	5917	6055	912 (15.1%)	2133 (35.2%)	3010 (49.7%)	867 (14.3%)
0.90	4948	5709	848 (14.9%)	1719 (30.1%)	3142 (55.0%)	1034 (18.1%)
1.35	4655	5529	1312 (23.7%)	1503 (27.2%)	2714 (49.1%)	820 (14.8%)
1.80	4628	5541	1218 (22.0%)	1355 (24.5%)	2968 (53.6%)	910 (16.4%)

Transport modes were assigned to signal packets identified from video analysis and grouped by sand feeding rate. The total number of packets comprises the number of packets associated with sliding or rolling motion and saltation, as well as the number of unclassified packets (i.e., $N^{corr}_{total} = N_{slide,roll} + N_{salt} + N_{uncl}$). Percentages are given relative to $N^{corr}_{total}$.

Considering individual particle-diameter classes (see Supplementary Fig. S2), the unclassified fraction in the video processing ranged from 5.6 to 36.6 % of particles when sand was present, depending on the gravel particle diameter. In contrast, under clear-water conditions, almost all particles smaller than 50 mm were successfully classified (0.7 % to 3.4 %). In the two largest particle diameter classes (50 and 60 mm), however, 23.3 % and 26.5 %, respectively, were not classified. These impacts occurred outside the camera’s observation window but still generated sufficient energy to produce apparent impacts in the acceleration signal.

### Signal response to sand transport

#### Spectral analysis

**Fig. 2:**
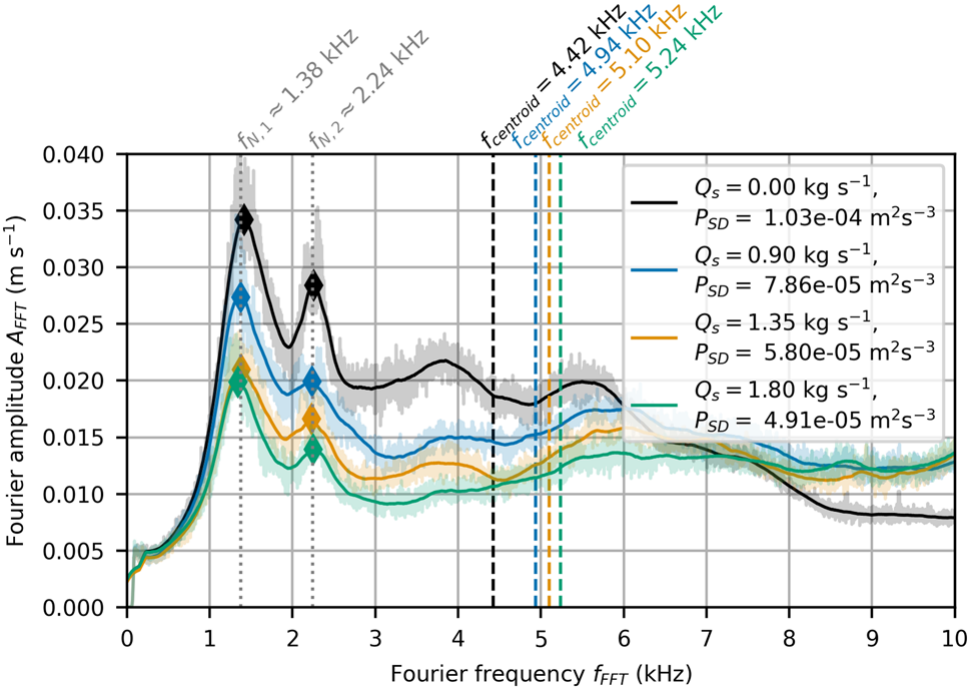
Fourier amplitude spectrum averaged over all experiments, showing the first and second natural frequencies ($f_{N,2}$, $f_{N,2}$) and the changes in the centroid frequency $f_{centroid}$ dependent on sand feeding rate $Q_s$. Frequency content below 0.1 kHz is filtered out during post-processing.

The median Fourier amplitude spectrum over one-second samples across 200 runs and 7 grain classes was calculated, resulting in a total of 1400 spectra with a spectral resolution of $10^{-3}$ kHz. Figure 2 displays the influence of sand on the spectral response by depicting the obtained amplitudes from a Fast Fourier Transform $A_{FFT}$ as a function of their corresponding Fourier frequencies $f_{FFT}$. It can be seen that increasing feeding rates led to an overall reduction in spectral density ($P_{SD}$) from $1.05\cdot 10^{-4}$ to $0.50\cdot 10^{-4}$ $\text {m}^2\text {s}^{-3}$. Additionally, the centroid frequency shifted from 4.41 kHz to 5.23 kHz, due to the suppression of amplitudes below approximately 6 kHz and a slight amplification of higher-frequency content ranging from 8 kHz to the sensor sample rate of 10 kHz. Furthermore, the first and second natural frequencies, $f_{N,1}$ and $f_{N,2}$, of the sensor system were identified at approximately 1.38 kHz and 2.24 kHz. Compared to clear water conditions, the amplitudes at $f_{N,1}$ reduced by 19.8, 36.4, and 41.7 %, and those at $f_{N,2}$ by 29.9, 42.0, and 51.3 %, for feeding rates of 0.90, 1.35, and 1.80 kg s$^{-1}$, respectively. Combined, these results demonstrate that the presence of sand inhibits the effect of coarse particle grain on the centroid frequency.

#### Analysis of packet properties

To assess the influence of the flow velocity and sand feeding rates on the registered 22,834 signal packets, representative statistics of the investigated properties, centroid frequency $f_{centroid}$, and maximum amplitude of the acceleration envelope $A^{max}_{env,acc}$, were calculated. These included the mean and coefficient of variation *σ*, and the skew *γ* of the probability distributions of packet properties. In addition to these summary statistics, we calculated the Jensen-Shannon (JS) divergence $D_{\text {JS}}$ to compare the similarity of the full probability distributions of packet properties obtained from experiments with and without sand feeding.

Therefore, we assessed the dependence of these characteristics on feeding rate and flow velocity. It shows the relative change of the mean $\Delta \mu _{rel}$ and $\Delta \sigma _{rel}$ with respect to clear water experiments, as well as the change in skew $\Delta \gamma$, as defined in the Supplementary Material. Furthermore, it shows the similarity of the probability distributions of sand-fed and clear water experiments based on the JS divergence $D_{\text {JS}}(Q_s=0\parallel Q_s\ne 0)$, which is a symmetric measure based on overlapping probability that becomes zero if these two are identical. A decrease in JS values would therefore emphasize that two distributions would become more similar, and vice versa.

**Fig. 3:**
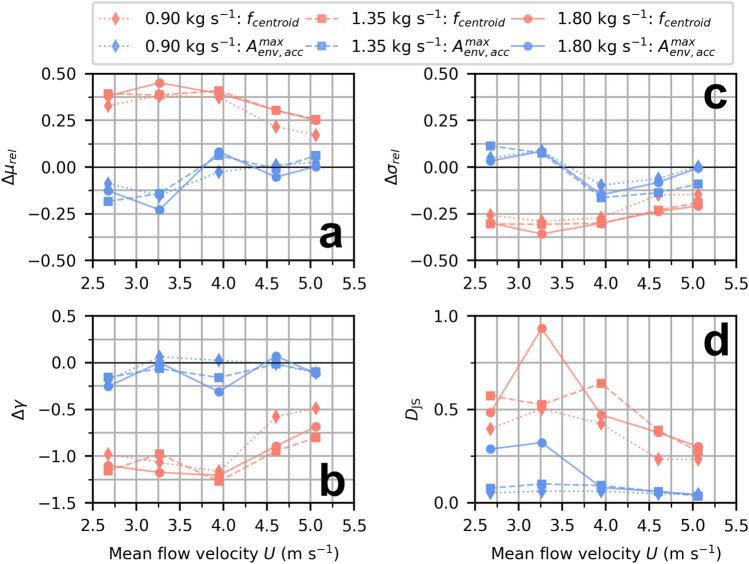
Statistics of signal packet properties, Centroid frequency $f_{centroid}$ and maximum amplitude of the acceleration envelope $A^{max}_{env,acc}$, across flow velocities and sand feeding rates. Shown are changes between sand-fed ($Q_s\ne 0$) and clear-water experiments ($Q_s=0$) in terms of mean $\Delta \mu _{rel}$ (**a**), skew $\Delta \gamma$ (**b**), and coefficient of variation $\Delta \sigma _{rel}$ (**c**). Additionally, JS divergence $D_{\text {JS}}$ (**d**) is evaluated between their probability distributions. Lower JS values signify higher similarity between the distributions.

Figure 3 shows that JS values for the centroid frequency and the maximum of the acceleration envelope across different flow velocities (2.7, 3.9, and 5.1 m s$^{-1}$) and sand feeding rates (0.90, 1.35, and 1.80 kg s$^{-1}$) are displayed. JS values closer to zero signify a higher similarity between the compared distributions.

It can be seen that the shape of the distribution of the frequency-based property changes considerably, even for the lowest sand feeding rate of 0.90 kg s$^{-1}$, the average JS value is 0.33, which increases to 0.43 and 0.46 for higher feeding rates of 1.35 and 1.80 kg s$^{-1}$, respectively. Also, the shape of the distributions changes notably, shifting from left-skewed (*γ =0.23*) to right-skewed (*γ =-0.79*). Furthermore, as seen previously in the analysis of frequency content, the mean of the centroid frequency for the packets shifts on average from 3.17 to 4.30 kHz.

The amplitude-based property, on the other hand, shows much more resilience towards the presence of sand, with a lower average JS value of 0.05, 0.07, and 0.10 for the same feeding rates. The average of $A^{max}_{env,acc}$ slightly decreases from 65.6 to 61.0 m s$^{-2}$ from the no-feeding experiment to the experiment with the maximum feeding rate, while the skew shifts right from 0.34 to 0.22.

From this, we can clearly see that sand primarily affects the frequency-based property related to the oscillatory response of the system, while the recorded maximum amplitudes remain nearly unchanged. This suggests that the sand does not reduce the amount of energy initially transmitted on impact.

Furthermore, the JS divergence of the packet properties shows a relationship to the flow velocity. This relationship is most pronounced for the centroid frequency $f_{centroid}$, where the JS value reduces from 0.47 to 0.23 when the flow velocity is increased from 2.7 to 5.1 m s$^{-1}$. The same, but less pronounced, trend is observed for $A^{max}_{env,acc}$, where the JS value reduces from 0.093 to 0.037. This indicates that less sand interacts with the sensor plates as an increasing share is transported in suspension.

### Particle diameter estimation

To further investigate the influence of sand on sensor response, signal packet properties were used to estimate the diameter of the corresponding particle class. To avoid signal attenuation effects, whereby apparent packets with small amplitudes dilute the average signal response associated with the actual particle diameter^28^, only packets generated by direct impacts identified through video analysis were considered.

The following evaluation focuses on two representative predictors that capture complementary aspects of the sensor response: an amplitude-based metric, the 85th quantile of the velocity envelope $A^{85th}_{env,vel}$, and a frequency-based metric, the centroid frequency $f_{centroid}$. The amplitude-based property was selected because it provided the most robust diameter estimates towards additional sand feeding in our analysis, in terms of Wald-test probability values. The centroid frequency was included for comparison, as it is a commonly used predictor in literature. Further analysis of packet properties and estimated power-law coefficients is provided in Supplementary Table S3, which also reports Wald-test probability values assessing the equality between regression lines obtained for sand-feeding experiments and the reference condition.

**Fig. 4:**
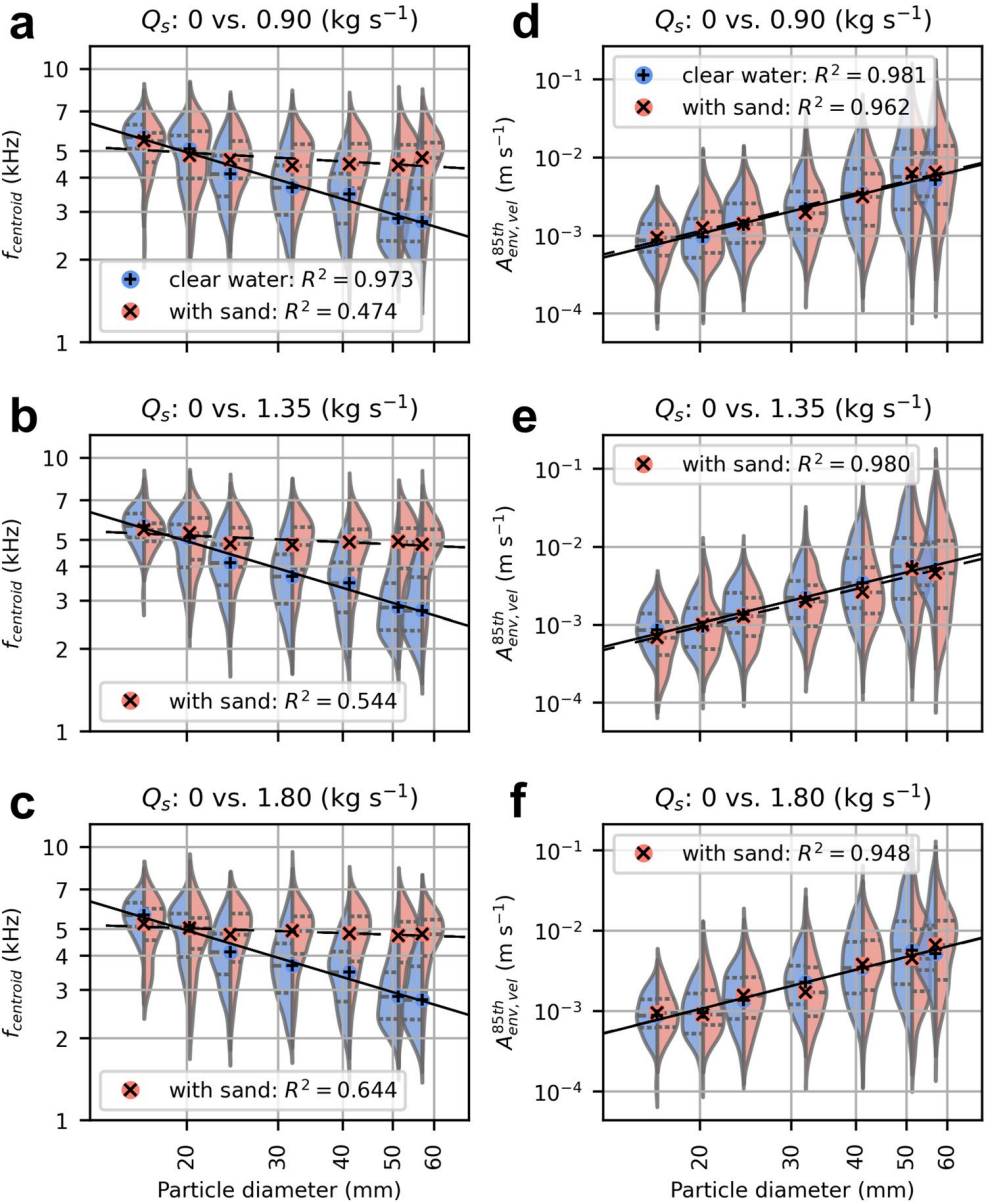
Power-law estimates for predicting mean diameters based on packet properties. Comparison between centroid frequency $f_{centroid}$ (**a,b**) and 85th quantile of velocity amplitudes $A^{85th}_{env, vel}$ (**d–f**) across sand-fed experiments with feeding rates of 0.90, 1.35, and 1.80 kg s$^{-1}$. Markers correspond to the median properties of packets classified as direct impacts on the sensor.

Figure 4 shows the regression lines for $A^{85th}_{env,vel}$ and for the centroid frequency $f_{centroid}$. The frequency-based predictor exhibits significant changes in slope and intercept, with Wald-test probability values below $10^{-3}$ for all feeding rates. Moreover, the estimated slope shows a slight negative trend with increasing feeding rate, indicating that the presence of sand produces packets with higher centroid frequencies.

Conversely, the amplitude-based predictor remains comparatively robust, with probability values of 0.14 to 0.67 for $A^{85th}_{env,vel}$. Additional amplitude metrics based on acceleration and displacement envelopes show similar agreement (mean probability over 0.25 across feeding rates). In contrast, other frequency-related properties, such as the frequency of maximum FFT amplitude and packet length, differ significantly (mean probability below 0.01, see Supplementary Table S2).

The root mean squared error (RMSE) was used to quantify the overall prediction error between the mean particle diameter of each class and the corresponding estimates. Because RMSE is sensitive to large deviations, the mean absolute percentage error (MAPE) was additionally evaluated to enable comparison across particle sizes. For $A^{85th}_{env,vel}$, sand feeding increased the RMSE from 2.52 to 3.28-3.97 mm and the MAPE from 5.0 to 8.8-9.2 %. In contrast, for $f_{centroid}$, the RMSE rose from 2.35 to 18.3-19.8 mm and the MAPE from 6.4 to 27.6-33.9 %, indicating a severe influence of sand on the frequency-based predictor. These results demonstrate that amplitude-based properties, in particular the velocity amplitude, are substantially more robust than the commonly used centroid frequency for inferring transported particle diameters.

### General influence on impulse and packet counts

As a first step, we assessed the variability of the number of registered impulses and packets, $N^{imp}$ and $N^{pack}$, respectively, by computing the coefficient of variability (*σ*) over each particle diameter class. The coefficient of variation is defined as the ratio of the standard deviation to the mean of a sample. It enables comparison of variability across particle size classes, as $N^{imp}$ and $N^{pack}$ are logarithmically distributed.

Especially for $N^{pack}$, the three smallest diameter classes (mean diameters smaller than 25 mm) exhibited the highest variability with *σ* ranging from 0.25 to 0.28. For larger diameters, the *σ* decreases constantly down to the largest diameter class with 0.13, nearly halving it. A similar but less pronounced trend is observed for $N^{imp}$, where the *σ* declines from 0.28 in the smallest diameter class to 0.21 in the largest (see Supplementary Material for further details). These results suggest that the number of repetitions for smaller particles may have been insufficient.

Further, we evaluated the relative differences in $N^{imp}$ and $N^{pack}$ between sand-fed and clear-water experiments, $\Delta N^{imp}$ and $\Delta N^{pack}$, respectively.

When evaluated with respect to the mean diameters of the seven investigated gravel particle classes, $\Delta N^{imp}$ shows a statistically significant, strong positive trend ($R^2 = 0.83$, $p = 4.7\cdot 10^{-8}$). On average, $\Delta N^{imp}$ increases from -18.8 to 50.6 % for mean diameters ranging from 16.5 to 56.9 mm, with an absolute average over all diameters of 29.5 %. Thus, the presence of sand reduced the number of registered impulses for smaller gravel particles, whereas it increased it for larger diameters. This behavior is likely due to the overlay of noise generated by the transported sand and the low-frequency oscillations following a particle impact. Since the duration of these oscillations increases with particle mass, their influence becomes more pronounced for larger diameter classes.

In contrast, $\Delta N^{pack}$ shows no significant trend with mean particle diameter. For the original packet counts, $\Delta N^{pack}$ varies between -24.9 and -5.9 %. The absolute average over all diameters resulted in 16.2 %. After correcting for overlapping packets, this range slightly reduces to -22.6 and +1.6 %, averaging at 8.3 %. Independent of the applied correction, the introduction of sand reduces the number of registered packets over all diameter classes.

The lower averaged $\Delta N^{pack}$ of 8.3 % and the absence of a diameter-dependent trend indicate that packet counts are comparatively more robust than impulse counts towards sand feeding. When further excluding the two smallest diameter classes with the highest coefficient of variation, the averaged $\Delta N^{pack}$ further reduced to 5.9 %, whereas the averaged $\Delta N^{imp}$ slightly increased to 32.2 %. This supports the claim of more robust packet counts.

Furthermore, we evaluated the differences $\Delta N^{imp}$ and $\Delta N^{pack}$ with respect to the mean flow velocity *U*. There, we found a statistically significant but weak relationship (*p=0.002*, $R^2 = 0.03$) between $\Delta N^{pack}$ and *U*. Averaged across all sand feeding rates, $\Delta N^{pack}$ decreases from -11.3 to -1.7 % as *U* increases from 2.7 to 5.1 m s$^{-1}$. This reduction indicates the declining effect of sand at higher flow velocity, as a greater fraction of sediment is transported in suspension.

A comparable, but less significant, trend is also observed for $\Delta N^{imp}$ (*p=0.011*, $R^2 = 0.35$). The relative difference declines from +35.7 % at the lowest velocity to +19.8 % at the highest. These consistently higher differences of $N^{imp}$ compared to $N^{pack}$, paired with weaker velocity dependence, further suggest that impulse counts are more strongly affected by sand transport than packet counts.

### Bedload transport rate estimations

To evaluate how the presence of sand affects the prediction accuracy of bedload discharge, we first investigated the experiment under clear-water conditions. A linear regression was fitted between the transported gravel mass per run and the corresponding number of registered impulses and packets. This yielded the mass–impulse and mass–packet coefficients, $k^{imp}_{b}$ and $k^{pack}_{b}$ respectively.

**Fig. 5:**
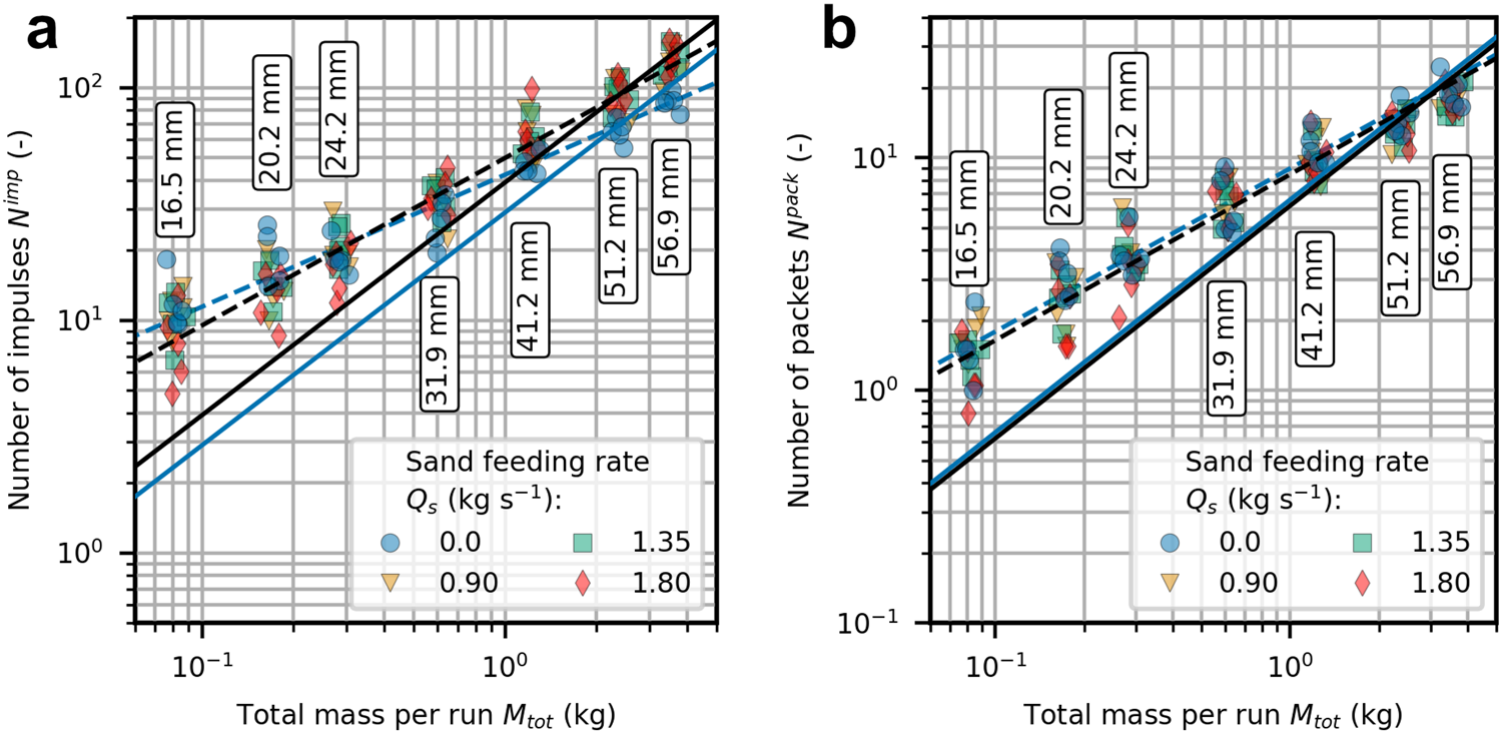
Number of registered impulses $N^{imp}$ (**a**) and packets $N^{pack}$ (**b**) as a function of total transported mass of gravel particles per run $M_{tot}$ across all experiments. Solid lines correspond to the linear relation derived from all experiments (*black*, with $k_b^{pack}=6.21$ kg$^{-1}$, and $k_b^{imp}=39.1$ kg$^{-1}$) or in clear water conditions only (*blue*, with $k_{b,q_s=0}^{pack}=6.60$ kg$^{-1}$, and $k_{b,q_s=0}^{imp}=29.0$ kg$^{-1}$). Dashed lines represent power-law relationships, respectively. Markers represent the mean of 10 repetitions conducted at the same flow velocity, while marker positions are horizontally jittered to improve visibility. The text box shows the mean particle diameter in (mm) of the corresponding sample of 15 gravel particles.

Figure 5 shows the number of registered impulses and packets, $N^{imp}$ and $N^{pack}$, respectively, as a function of total gravel mass passed per experimental run $M_{tot}$. As each run consists of the same number of gravel particles, $M_{tot}$ directly relates to the mean diameter of the particle class. It further displays the OLS regression between these variables, with the vertical position indicating the fitted slope, i.e., $k^{imp}_{b}$ and $k^{pack}_{b}$.

This relationship reveals that gravel particle diameters smaller than 25 mm result in a disproportionate increase of $N^{imp}$ and $N^{pack}$, indicating the presence of a bias. A likely explanation is that, together with the small number of particles transported per run, the selected threshold value of 1 g may be too permissive. This allows even a single instance of ambient noise exceeding the threshold to introduce a noticeable bias in the recorded number of impulses and packets.

As a next step, we used the fitted coefficients, $k^{imp}_{b}$ and $k^{pack}_{b}$, to evaluate bedload discharge for all experimental runs, including those with sand feeding. The resulting error metrics were then compared with error metrics derived from regressions calibrated on all experiments, i.e., clear water and sand-fed.

Regarding impulse-based predictions, $k^{imp}_{b}$ increases substantially from 29.0 kg$^{-1}$ ($R^2 = 0.94$) to 39.1 kg$^{-1}$ ($R^2 = 0.93$), when sand-fed experiments are considered in the calibration. This change manifests in the reduction of the RMSE evaluated across all experiments of almost 40% from 0.714 to 0.434 kg. Furthermore, the MAPE reduces from 128.5 % to 77.1 %.

In contrast, for packet-based predictions, the inclusion of sand-fed experiments alters the derived $k^{pack}_{b}$ slightly, decreasing it from 6.60 kg$^{-1}$ ($R^2 = 0.93$) to 6.21 kg$^{-1}$ ($R^2 = 0.93$). Consequently, the resulting error metrics remain largely stable, as the RMSE decreases slightly from 0.464 to 0.454 kg. Notably, the MAPE increases from 78.2 % to 86.1 %. This contradiction is explained by the linear regression being fitted to optimize the absolute RMSE metric, which does not align with optimizing the relative error metric.

Interestingly, the calibration based on all experiments leads to an equal, even slightly better, performance of impulse-based bedload discharge predictions, evident in both RMSE (0.434 kg vs. 0.454 kg) and MAPE (77.1% vs. 86.1%). This result indicates that both methods are suitable predictors if sand feeding is properly included in the calibration process.

Additionally, we evaluated prediction accuracy using a power-law relationship instead of a linear model. Consistent with previous observations, the packet-count-based model demonstrates greater robustness to the presence of sand. The RMSE changes only marginally, from 0.455 kg to 0.411 kg. In contrast, the impulse-based model shows a notable reduction in RMSE, from 1.66 kg to 0.486 kg, when sand-fed experiments are included in model calibration. A similar trend is observed for MAPE. The packet-based model exhibits only a minor change (26.6 % to 27.9 %), whereas the impulse-based model improves, with MAPE decreasing from 49.5 % to 29.7 %.

## Discussion

The present results demonstrate that sand transport fundamentally alters the vibrational response of impact-plate monitoring systems based on accelerometers and therefore cannot be neglected when deriving flume-based calibrations of these systems. Rather than reflecting changes in the impact process of gravel particles, the observed spectral attenuation appears to arise from the additional mass of sand acting on the sensor plate. This argument is supported by comparing packet statistics with the general spectral analysis: Despite a pronounced reduction in the Fourier amplitudes at lower frequencies and a halving in spectral density from $1.03\cdot 10^{-4}$ to $0.49\cdot 10^{-4}$ m$^{-2}$s$^{-3}$, the maximum amplitudes of the impact-related packets decreased only marginally, indicating that the transmitted energy on impact remains nearly constant. The observed attenuation further reduces with increasing flow velocity, as a larger portion of sand is transported in suspension rather than as bedload, and the effective sand mass acting on the plate diminishes. A decreasing JS divergence between sand-fed and clear-water packet property distributions likewise indicates a reduced sand influence with increasing flow velocity.

This altered signal response severely reduces the robustness of the packet-based diameter estimation. This is most evident in the significant change of the power law relationship with $f_{centroid}$. The Wald test p-value falls below $1\cdot 10^{-3}$, rejecting equivalence with clear-water conditions, and the correlation coefficient declines drastically from 0.973 to 0.495. This also manifests in the increase of the MAPE from 6.4 to 33.9 % when sand is fed. This sensitivity is problematic as Nicollier et al. (2022)^28^ based the upper threshold of a diameter class on the combination of $A^{max}_{env,acc}$ and $f_{centroid}$, whereas Wyss et al. (2016)^27^ used the same packet properties to predict sample mean diameters.

In contrast, amplitude-based properties demonstrate considerably greater robustness towards the presence of sand. Specifically, the power-law fits of $A^{85th}_{env,vel}$ remains statistically equivalent to clear-water conditions (Wald $p=0.14 \text{ to } 0.90$) and maintains strong correlations ($R^2>0.94$). This contrast indicates that sand primarily affects frequency-related properties, representing the system’s oscillating response, more than amplitudes, which are linked to impact energy transfer.

Sand also introduces a systematic bias in gravel-transport estimates derived from impulse counts. The large change in the number of registered impulses during sand feed experiments compared to clear-water conditions, evident in a $\Delta N^{imp}$ of 29.5 %, implies that impulse-based methods would consequently overestimate true gravel transport when sand is transported as well.

Packet counts, however, remain more stable ($\Delta N^{imp}\approx 5.9~\%$), supporting the assumption that packets represent individual particle–plate interactions and therefore are a more robust regressor for gravel-sized transport. Importantly, this only occurs after correcting the number of packets to account for overlapping impacts. The applied correction relies on a parameter calibrated exclusively using clear-water experiments, but it produces a disproportionately high number of split packets when sand is present. This circumstance is reasonably explained by the additional noise generated by the sand, which facilitates threshold exceedance during signal packet segmentation. Nonetheless, this parameter can also serve as a calibration variable, since adjusting it equalizes the number of registered packets in experiments with and without sand feeding.

Potential differences in particle impact probability were also considered. Although impact likelihood depends on jump length, distance between the introduction point of the gravel and the sensor, as well as dominating transport mode, neither video observations nor experimental controls indicated systematic differences between sand-fed and clear-water runs: identical particles were released from the same upstream position, and no clear change in transport mode was detected.

The comparison between mass–impulse and mass–packet coefficients, $k^{imp}$ and $k^{pack}$, calibrated exclusively on clear-water experiments and on all experiments, highlights the sensitivity of impulse-based predictions to the presence of sand, whereas packet-based predictions appear more robust. When sand-fed experiments are incorporated into the calibration, the impulse coefficient increases markedly, leading to a substantial reduction in RMSE of almost 40 %. This indicates that impulse counts are strongly influenced by the presence of sand, and neglecting these conditions in the calibration process can bias discharge estimates. In contrast, the packet-based coefficient changes only marginally, and the corresponding error metrics remain relatively stable.

Overall, the results demonstrate that packet-based predictions are less sensitive to calibration conditions, whereas impulse-based predictions can only achieve comparable accuracy when sand transport is explicitly included in the calibration process.

The experimental conditions of this study were conducted with high flow velocities, a disproportionately large fraction of transported sand relative to gravel, and relatively low transport rates compared to the estimated capacity (maximum 9.9 %). Such conditions are typically encountered during flushing and sluicing operations at run-of-river hydropower plant weirs, and therefore represent a specific case rather than natural equilibrium transport conditions.

Consequently, the application of the findings to natural mountainous streams and gravel-bed rivers is limited. In particular, the intentionally low gravel transport rates excluded particle interaction almost entirely, though they are an essential part of natural transport processes influencing entrainment, impact probabilities, and transport modes. Moreover, natural rivers typically convey a wide range of sediment sizes, including variable sand fractions, whereas this study focused on a narrow range of coarse sand (1 to 2 mm). As a result, the transferability of the results to finer or coarser fractions, approaching the lower detection limit of impact-plate systems, remains uncertain. Since grain size directly influences impact energy and entrainment thresholds, the quality of fed sediments will certainly affect the sensor response.

Nevertheless, the observed qualitative alteration in the signal response of the impact plates is likely to persist under natural conditions, specifically a shift toward higher-frequency vibrations due to the damping effects of the interacting sand. However, quantitative calibration remains site-specific.

Future studies should therefore investigate higher gravel transport rates to capture these particle interactions and to evaluate the robustness of the presented findings.

Furthermore, the transferability of these results, obtained using accelerometers, to plate systems employing different sensor types (e.g. geophones) was not particularly investigated and requires further investigation using combined measurement approaches. This is particularly important because accelerometers are known to be sensitive to higher-frequency vibrations, such as those introduced by sand. Using both systems offers a complementary approach, allowing their individual strengths to be combined.

A further source of uncertainty is the high variability observed in the number of impulses and packets for particle size classes smaller than 25 mm. This high variability is not strictly a hardware limit, but arises mainly from the lower impact probability of smaller gravel particles on the sensor plate due to the larger jump length associated with smaller particles. Another factor is the limited number of particles tested per run. According to the experimental design, the number of particles per class was fixed at 15. This resulted in 300 passes per particle class over both sensors. This setup was chosen to prioritize the tracking of single-particle trajectories, especially under conditions of high sand feeding rates, while balancing the increasing demand for sand. Handling such large amounts of sand in laboratory experiments remains challenging and could potentially be mitigated by using a recirculating setup instead of the sediment feeding approach used in this study. Nevertheless, adopting a recirculating setup would introduce additional complexities, notably the unsteady sediment supply that is characteristic of such systems.

 This limitation in the number of particles tested further affects the number of registered impulses and packets for the smallest particle diameters. Under these conditions, even a single instance of ambient noise exceeding the threshold is sufficient to introduce a noticeable bias in the recorded impulses and packets.

## Conclusion

Impact plate systems are commonly used to monitor gravel-sized bedload transport in mountainous streams, rivers, and sediment bypass tunnels. The commonly used sensor types include geophones, microphones, and, as investigated in this study, accelerometers. However, the influence of sand transport on these sensors remains largely unexamined. This study demonstrates that sand transport considerably affects the sensor responses to gravel-sized particle impacts. This aspect has received little attention in efforts to derive global calibrations from controlled laboratory experiments.

By analyzing the nonparametric statistics of the spectral properties of signal packets, we show that the interaction of sand with an impact plate accelerometer has a non-negligible influence on its signal response to particle impacts. The introduced interferences lead to significant misestimations of particle size based on frequency properties, whereas amplitude-based properties are far less affected. Consequently, when sand is transported, diameter prediction should be based exclusively on amplitude characteristics, not on the widely used combination of frequency and amplitude.

Further analysis of statistical mass transport estimates reveals that packet-count-based mass ratios are more robust than those derived from impulse counts, provided that overlapping packets are properly accounted for. This adjustment introduces an additional calibration parameter that regulates the packet count and reduces the initial difference between sand-fed and clear-water results in this study by nearly two-thirds. Future statistical transport predictions should therefore prioritize packet-based approaches over impulse-based ones to improve robustness against sand-induced noise.

These insights address a critical limitation in efforts to derive a global calibration for impact plate systems. They underscore the need to more closely replicate natural conditions in laboratory settings, highlighting the importance of sensor–sand interactions. The resulting improvement in bedload monitoring accuracy not only enhances our understanding of fluvial bedload transport but also supports a more effective management of riverine ecosystems.

## Supplementary Information

Supplementary Information.

## Data Availability

Data and code is available on Zenodo at https://doi.org/10.5281/zenodo.17799396
