# Supplementary material for: The role of sand in an impact-based bedload monitoring system
Source: Sci Rep. 2026 May 26;16:23973. doi: 10.1038/s41598-026-53231-x (PMC13434743; doi:10.1038/s41598-026-53231-x)
Supplement: Supplementary file 1 [file 41598_2026_53231_MOESM1_ESM.pdf]

Supplementary material: The role of sand in an impact-based bedload monitoring system

Manuel Pirker[1,*], Hannes Badura[2], Josef Schneider[1], and Stefan Haun[1]

[1]Graz University of Technology, Institute of Hydraulic Engineering and Water Resources Management, Stremayrgasse 10/II, Graz, 8010, Austria
[2]VERBUND Hydro Power GmbH, Europaplatz 2, 1150 Vienna, Austria
[*]manuel.pirker@tugraz.at

Hydraulic parameters

The mean flow velocity in $(\mathrm{m\ s^{-1}})$ is calculated as the average during the recording time of each run within the section of the impact sensor at the outlet of the flume by:

$$\mathrm{U} = \frac{\overline{Q}}{\overline{h} \cdot b} \tag{1}$$

Where $\overline{Q}$ is the measured discharge at the inlet of the flume, averaged over the recording time, $\overline{h}$ is the averaged water depth above the sensors, and $b = 0.20$ m is the constant width of the flume.

In a similar manner, Froude number Fr and Reynolds number Re are derived:

$$\mathrm{Fr} = \frac{\overline{Q}}{\overline{h}^{1.5} \cdot b \cdot \mathrm{g}^{0.5}} \tag{2}$$

$$\mathrm{Re} = \frac{4 \cdot \overline{Q} \cdot \overline{r}_{\mathrm{hyd}}}{\overline{h} \cdot b \cdot \nu} \tag{3}$$

Where $\mathrm{g} = 9.81\ \mathrm{m\ s^{-1}}$ is the gravitational constant, $\overline{r}_{\mathrm{hyd}}$ is the hydraulic radius averaged over the recording time, and ν is the kinematic viscosity calculated for each run based on the measured water temperature.

Flow regime

The shear velocity U^* in $(\mathrm{m\ s^{-1}})$ is calculated with the Darcy friction factor f using Chen's approximation[1]. The friction factor accounts for the combined friction of the flume's concrete bed and glass walls:

$$f = \frac{f_{\mathrm{bed}} \cdot b + 2 \cdot f_{\mathrm{wall}} \cdot \overline{h}}{b + 2 \cdot \overline{h}}$$

$$U^* = \left(\frac{1}{8} \cdot f \cdot U^2\right)^{0.5}$$

Finally, the roughness Reynolds number k_s^+ is obtained by:

$$k_s^+ = \frac{U^* \cdot k_s}{\nu}$$

Where $k_s = 0.175 \cdot 10^{-3}$ m is the sand grain roughness obtained by fitting a backwater curve to water level measurements along the flume during stationary conditions.

Transport capacity

The unit transport capacity of the feeding sand q_s is calculated with the corrected Meyer-Peter-Müller formula[2]:

$$q^* = 4.93 \cdot (\tau_b^* - 0.047)^{1.6}$$

$$q^* = \frac{\hat{q}_s}{\sqrt{R \cdot g \cdot D_m^3}}$$

Where q^* is the dimensionless transport capacity, $R = 1.65$ is the submerged specific gravity of the feeding sand, $D_m = 0.0015$ m is the mean diameter of feeding sand, and τ_b^* is the dimensionless bed shear stress calculated from the shear velocity:

$$\tau_b = \frac{U^{*2}}{r_{\text{hyd,b}} \cdot g \cdot D_m}$$
$$\tau_b^* = \frac{\tau_b}{\rho \cdot R \cdot g \cdot D_m}$$

With $r_{\text{hyd,b}}$ is the reduced hydraulic radius accounting for the effect of the flume's side walls.

Diameter-mass relation

The diameter-mass relation was derived by fitting a power law, resulting in the following gravel particle diameter estimate D_g in (m) given the particle mass G_g in (kg):

$$D_g = 9.42 \cdot 10^{-2} \cdot G_g^{0.338}, \quad R^2 = 0.975 \tag{4}$$

Video analysis

The impact location for saltation particle was derived from the position of the gradient breakpoint in a two-segmented piecewise regression model[3] fitted to the particle trajectory:

$$y = \alpha_1 \cdot x + (\alpha_2 - \alpha_1) \cdot (x - x_b) \cdot H(x - x_b) + c + \eta, \tag{5}$$

where y and x are the centroid coordinates of the contour, α_1 and α_2 are the incident and reflected trajectory segments, respectively, x_b is the location of the breakpoint, H is the Heaviside step function, c is a constant offset, and η is a noise term. Finally, the time difference between the registered packet and the impact in video, $\Delta t^{impact}_{signal,video}$, was derived from the distance between the breakpoints x_b and the horizontal centroid location of the particle contour at the time the packet was recorded, x_t:

$$\Delta t_{sig,vid} = \frac{x_b - x_t}{v_{p,x}}, \tag{6}$$

where $v_{p,x}$ is the particle velocity in flow direction. Impacts were counted as valid if the breakpoint lay within the boundaries of the two sensors and the time difference was smaller than 13.8 milliseconds from the time the packet was recorded. This threshold corresponds to one standard deviation of the temporal offsets of all impacts. Figure S1 shows the tracked trajectories of a representative particle with a diameter of 30 mm under either clear water conditions or during the highest sand feeding rate.

Table S2 shows the cumulative mass of sand fed during the experiments, depending on the mass feeding rate. Furthermore, Figure S2 shows the transport modes identified via video analysis per diameter class and feeding rate.

Error metrics

The mean absolute error (MAE) and the mean squared error (RMSE) are evaluated for estimations for gravel particle diameters and mass transport rates. They are defined in terms of the errors between estimated $\hat{y}$ and the measured values y:

$$\text{MAE} = \frac{1}{N} \sum_i^N |\hat{y}_i - y_i| \tag{7}$$

$$\text{RMSE} = \sqrt{\frac{1}{N} \sum_i^N (\hat{y}_i - y_i)^2} \tag{8}$$

Additionally, the mean absolute percentage error (MAPE) is evaluated, which emphasizes proportional error towards the actual measured values:

$$\text{MAPE} = \frac{1}{N} \sum_i^N \left| \frac{\hat{y}_i - y_i}{y_i} \right| \tag{9}$$

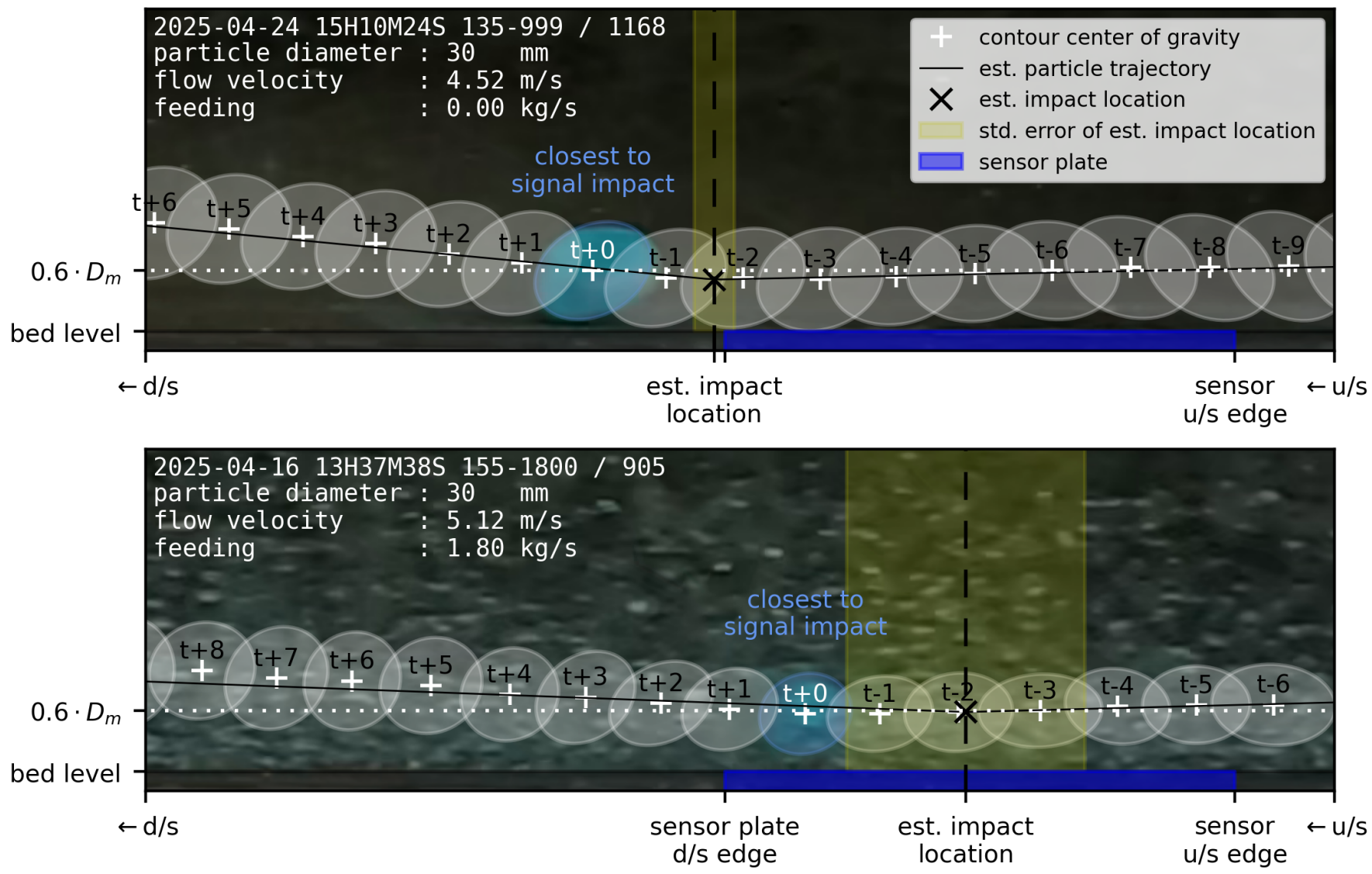

Figure S1. Identified trajectories from the contour tracker of a 30 mm diameter particle within clear water conditions (top), and with maximum sand feeding rate at 1.80 kg s^{-1} (bottom).

Coefficient of variation

The coefficient of variation[4] (CV) shows the variability in terms of the standard deviation s relative to its mean μ, and is defined as:

$$\sigma = \frac{s}{\mu} \tag{10}$$

It allows comparison of variability across samples of different scales and units.

Relative statistics

The relative changes in the mean and coefficient of variation with respect to clear-water experiments were computed as:

$$\Delta\mu_{rel} = \frac{\mu - \mu_{Q_s=0}}{\mu_{Q_s=0}} \tag{11}$$

$$\Delta\sigma_{rel} = \frac{\sigma - \sigma_{Q_s=0}}{\sigma_{Q_s=0}} \tag{12}$$

Where μ and σ denote the mean and coefficient of variation of the distribution of all packets registered at a given flow velocity and sand feeding rate. The reference quantities $\mu_{Q_s=0}$ and $\sigma_{Q_s=0}$ correspond to the respective measures obtained at the same flow velocity under clear-water conditions ($Q_s = 0$).

The absolute change in skewness $\Delta\gamma$ is computed as:

$$\Delta\gamma = \gamma - \gamma_{Q_s=0} \tag{13}$$

Where γ represents the skewness of the packet-property distribution at a given flow velocity and sand feeding rate, and $\gamma_{Q_s=0}$ denotes the corresponding skewness under clear-water conditions.

Jensen-Shannon distance

The Jensen-Shannon distance (JS) is a statistical distance measure of how much a probability distribution P(x) differs from another probability distribution Q(x). It is obtained by the combination of the Kullback-Leibler divergences (KL) of the two distributions to retrieve a symmetrical measure. JS is always positive, and higher values indicate a higher difference, while a JS value of zero indicates identity. The discrete case is defined as follows:

$$D_{\mathrm{JS}}(Q_s = 0 \parallel Q_s \neq 0) = 0.5 \cdot \left[D_{\mathrm{KL}}(P \parallel Q) + D_{\mathrm{KL}}(Q \parallel P)\right], \tag{14}$$

$$\text{with } D_{\mathrm{KL}}(P \parallel Q) = \sum_{x \in X} p(x) \log\left(\frac{p(x)}{q(x)}\right) \neq D_{\mathrm{KL}}(Q \parallel P) \tag{15}$$

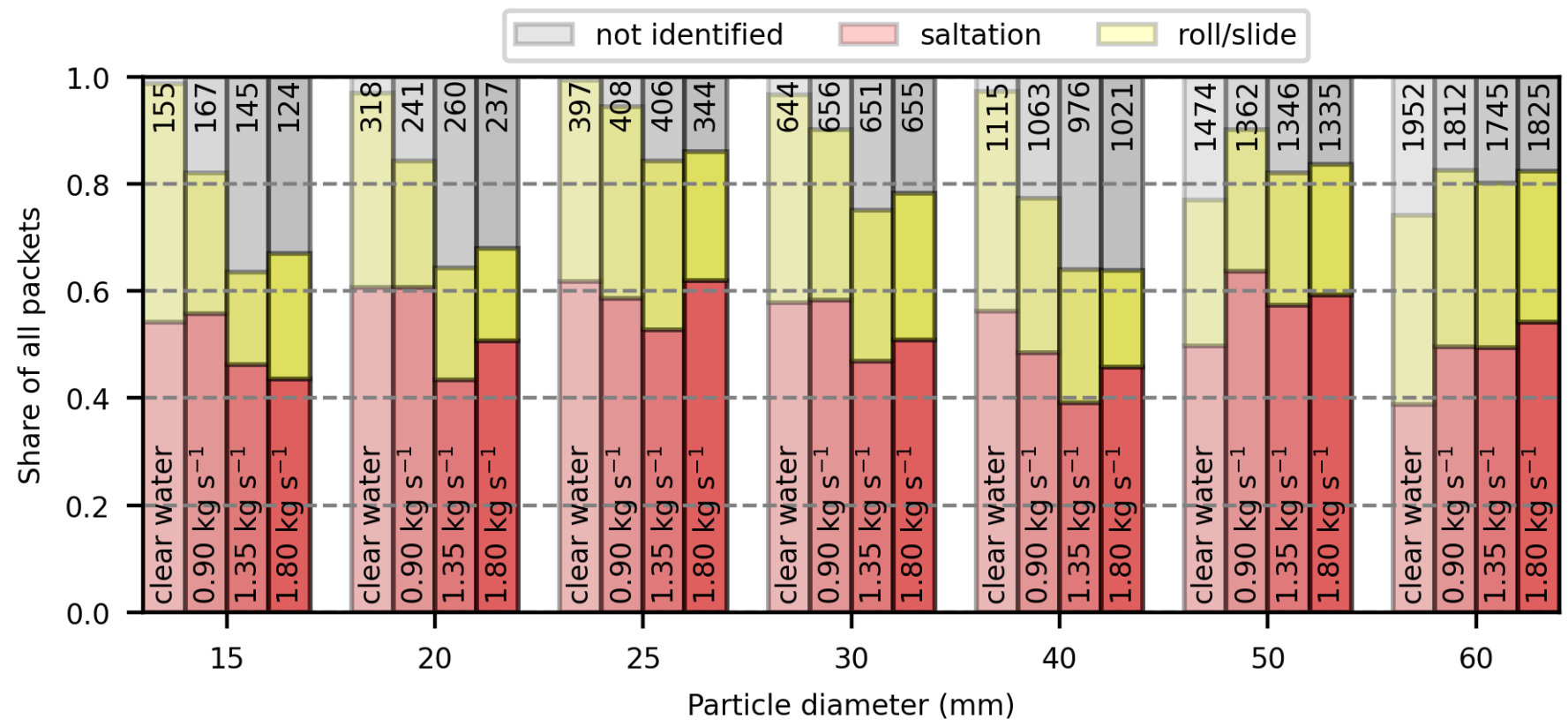

Figure S2. Particle transport modes per velocity and diameter. Transport modes of the gravel particles were identified from video analysis, aggregated over all investigated flow velocities for each sand feeding rate and particle diameter class. The number of detected particles in the video clip is displayed at the top of each column.

Signal packet diameter estimation

Statistical results for the properties with the highest Wald-test values are shown in Table S1. These properties are derived for each registered packet individually. They include quantile values of the envelope amplitude for acceleration, velocity and displacement ($A^{95th}_{env,acc}$, $A^{85th}_{env,vel}$, and $A^{75th}_{env,disp}$), the integral of the absolute acceleration (I), the maximum amplitude of the FFT amplitude spectrum (A^{FFT}_{max}), the packet length (L_{pack}), and the centroid frequency ($f_{centroid}$).

Mass rate estimation

Table S3 presents the differences in impulse and packet counts relative to clear water conditions, ΔN^{imp} and ΔN^{pack}, for each mean gravel particle diameter. For ΔN^{imp}, the positive slope of 0.018 indicates that impulse counts decrease as flow velocity increases. This relationship is highly statistically significant ($R^2 = 0.834$, $p = 4.7 \cdot 10^{-8}$). In contrast, packet counts exhibit no significant trend in association with mean particle diameter.

Table S4 presents the differences in impulse and packet counts relative to clear water conditions, ΔN^{imp} and ΔN^{pack}, for each investigated flow velocity setting. For ΔN^{imp}, the negative slope of -0.071 indicates that impulse counts decrease as flow velocity increases. This relationship is statistically significant ($p = 0.011$, $R^2 = 0.354$). In contrast, packet counts exhibit a positive association with flow velocity, with a slope of 0.037, which is highly significant ($p = 0.00185$, $R^2 = 0.503$).

Table S5 shows the error metrics determined from the predicted mass transport rates derived from all experiments' feeding rates for the linear relationship between counts and mass. When the model is calibrated using only clear-water experiments, the resulting linear relationship is given by:

$$M_g = \frac{1}{29.0} \cdot N^{imp} \qquad R^2 = 0.937 \qquad (16)$$

$$M_g = \frac{1}{6.60} \cdot N^{pack} \qquad R^2 = 0.930 \qquad (17)$$

Considering all experiments:

$$M_g = \frac{1}{39.1} \cdot N^{imp} \qquad R^2 = 0.934 \qquad (18)$$

$$M_g = \frac{1}{6.21} \cdot N^{pack} \qquad R^2 = 0.928 \qquad (19)$$

Where M_g is the mass of the gravel particle sample, N^{imp} is the number of impulses registered during its passage, and N^{pack} is the number of packets.

Additionally, Table S6 shows the same error metrics computed using a power-law relationship between counts and mass.

The power law derived only from clear water experiments ($Q_s = 0$) is:

$$M_g = 0.00136 \cdot (N^{imp})^{1.763}, \qquad R^2 = 0.919 \tag{20}$$

$$M_g = 0.0436 \cdot (N^{pack})^{1.427}, \qquad R^2 = 0.937 \tag{21}$$

(22)

The power-law relationship derived from the full set of experiments is given by:

$$M_g = 0.00438 \cdot (N^{imp})^{1.390}, \qquad R^2 = 0.924 \tag{23}$$

$$M_g = 0.0500 \cdot (N^{pack})^{1.402}, \qquad R^2 = 0.933 \tag{24}$$

(25)

References

1. Chen, N. H. An explicit equation for friction factor in pipe. *Ind. Eng. Chem. Fund.* **18**, 296–297, DOI: 10.1021/i160071a019 (1979).

2. Wong, M. & Parker, G. Reanalysis and correction of bed-load relation of meyer-peter and müller using their own database. *J. Hydraul. Eng.* **132**, 1159–1168, DOI: 10.1061/(ASCE)0733-9429(2006)132:11(1159) (2006).

3. Pilgrim, C. Piecewise-regression (aka segmented regression) in Python. *J. Open Source Softw.* **6**, 3859, DOI: 10.21105/joss.03859 (2021).

4. Everitt, B. *The Cambridge dictionary of statistics* (Cambridge University Press, Cambridge, 1998).

Property	Sand mass feeding rate Q_s (kg s^{-1})	OLS estimate log(a)	b	R^2	Wald test W_{θ_0,θ_1}	Error estimates MAE (mm)	RMSE (mm)	MAPE (%)
$A^{85th}_{env,vel}$ (m s^{-1})	0	-5.10	1.63	0.981		1.81	2.53	5.0
	0.90	-5.03	1.61	0.962	0.22	3.12	3.91	8.8
	1.35	-5.10	1.60	0.980	0.14	3.13	3.97	8.2
	1.80	-5.08	1.62	0.948	0.67	2.98	3.28	9.2
$A^{85th}_{env,disp}$ (m)	0	-8.57	1.60	0.977		2.15	2.63	6.2
	0.90	-8.57	1.59	0.977	0.80	2.10	2.71	5.4
	1.35	-8.58	1.54	0.977	7.7e-03	5.11	6.13	14.5
	1.80	-8.47	1.50	0.924	0.23	3.30	4.62	10.2
I (m s^{-1}·s)	0	-3.14	1.10	0.952		2.64	3.41	8.5
	0.90	-3.41	1.28	0.888	0.07	6.77	9.03	17.0
	1.35	-3.38	1.23	0.908	0.25	5.80	6.77	16.4
	1.80	-3.58	1.37	0.889	0.15	5.40	7.44	16.1
$A^{95th}_{env,acc}$ (m s^{-2})	0	-0.43	1.26	0.974		2.43	3.02	6.7
	0.90	-0.34	1.19	0.901	0.63	3.75	4.75	11.3
	1.35	-0.15	1.04	0.917	0.02	4.62	5.62	13.5
	1.80	-0.37	1.18	0.886	0.17	4.14	5.28	13.3
A^{FFT}_{max} (m)	0	-1.21	1.21	0.939		3.65	5.02	10.0
	0.90	-1.16	1.12	0.884	0.08	4.93	6.50	14.0
	1.35	-1.17	1.09	0.955	2.3e-03	8.10	9.29	22.1
	1.80	-1.28	1.17	0.910	8.5e-03	7.33	8.29	20.9
L_{pack} (-)	0	1.13	0.53	0.987		1.20	1.29	4.4
	0.90	0.96	0.68	0.929	1.3e-04	13.83	21.68	31.1
	1.35	1.01	0.63	0.929	6.8e-04	7.80	13.72	17.4
	1.80	1.05	0.62	0.918	2.2e-04	11.58	17.50	28.2
$f_{centroid}$ (kHz)	0	4.43	-0.57	0.973		2.04	2.35	6.4
	0.90	3.83	-0.11	0.474	4.8e-04	12.85	18.25	27.6
	1.35	3.82	-0.08	0.544	1.1e-05	15.01	20.12	33.9
	1.80	3.78	-0.06	0.644	7.2e-07	14.75	19.75	33.4

Table S1. Power law estimates a and b for the OLS model to predict particle diameter by signal packet properties. The correlation coefficient (R^2) shows the linearity of fit. Statistical test p-values show the significance of the difference in clear water conditions.

sand feeding rate kg s^{-1}	unit mass rate kg m^{-1}s^{-1}	feeding time per class s	mass per run kg	repetitions	total mass kg
0.90	4.50	5	23	50	1150
1.35	6.75	5	47	50	2362
1.80	9.00	5	63	50	3150

Table S2. Summary of coarse sand feeding material.

Mean particle diameter D_m (mm)	sand mass feeding rate Q_s (kg s^{-1})	rel. differences impulse counts ΔN^{imp}	rel. differences packet counts ΔN^{pack}
56.9	0.90	+0.463	-0.072
	1.35	+0.498	-0.106
	1.80	+0.557	-0.065
51.2	0.90	+0.421	-0.076
	1.35	+0.465	-0.087
	1.80	+0.452	-0.094
41.2	0.90	+0.361	-0.047
	1.35	+0.320	-0.125
	1.80	+0.418	-0.084
31.9	0.90	+0.193	+0.019
	1.35	+0.270	+0.011
	1.80	+0.294	+0.017
24.2	0.90	+0.085	+0.028
	1.35	+0.152	+0.023
	1.80	-0.111	-0.134
20.2	0.90	-0.235	-0.242
	1.35	-0.241	-0.182
	1.80	-0.332	-0.255
16.5	0.90	-0.082	+0.077
	1.35	-0.140	-0.065
	1.80	-0.323	-0.200

Table S3. Relative differences in impulse (ΔN^{imp}) and packet (ΔN^{pack}) counts compared to clear water conditions, presented for each combination of mean gravel particle diameter D_m and sand feeding rate Q_s. Positive values indicate an increase relative to clear water, while negative values indicate a decrease.

Mean flow velocity U ($m\ s^{-1}$)	sand mass feeding rate Q_s ($kg\ s^{-1}$)	rel. differences impulse counts ΔN^{imp}	rel. differences packet counts ΔN^{pack}
2.7	0.90	+0.342	-0.104
	1.35	+0.326	-0.122
	1.80	+0.404	-0.112
3.3	0.90	+0.444	-0.043
	1.35	+0.349	-0.118
	1.80	+0.353	-0.105
3.9	0.90	+0.447	-0.030
	1.35	+0.452	-0.103
	1.80	+0.418	-0.131
4.6	0.90	+0.189	-0.089
	1.35	+0.292	-0.030
	1.80	+0.295	-0.037
5.1	0.90	+0.125	-0.002
	1.35	+0.264	-0.033
	1.80	+0.204	-0.016

Table S4. Relative differences in impulse (ΔN^{imp}) and packet (ΔN^{pack}) counts compared to clear water conditions, presented for each combination of mean flow velocity U and sand feeding rate Q_s. Positive values indicate an increase relative to clear water, while negative values indicate a decrease.

	Sand mass feeding rate Q_s ($kg\ s^{-1}$)	MAE $\varepsilon_{Q_s=0}$ (kg)	MAE ε (kg)	MAE $\Delta\varepsilon$ (%)	RMSE $\varepsilon_{Q_s=0}$ (kg)	RMSE ε (kg)	RMSE $\Delta\varepsilon$ (%)	MAPE $\varepsilon_{Q_s=0}$ (%)	MAPE ε (%)	MAPE $\Delta\varepsilon$ (%)
Packets	0	0.370	0.391	+5.57	0.450	0.461	+2.51	87.8	97.0	+10.4
	0.90	0.377	0.374	-1.00	0.470	0.459	-2.47	82.6	90.4	+9.44
	1.35	0.374	0.371	-0.70	0.483	0.461	-4.43	77.8	85.4	+9.78
	1.80	0.339	0.336	-0.92	0.453	0.435	-3.87	64.7	71.7	+10.8
Impulses	0	0.379	0.429	+13.0	0.424	0.598	+41.1	134.7	91.4	-32.2
	0.90	0.619	0.309	-50.1	0.770	0.387	-49.7	135.0	79.9	-40.8
	1.35	0.638	0.291	-54.4	0.762	0.335	-56.1	134.1	77.7	-42.1
	1.80	0.641	0.274	-57.2	0.829	0.364	-56.1	110.0	59.3	-46.1

Table S5. Comparison of error metrics for predicted mass transport rates derived from all experiments feeding rate ε (with $k_b^{pack} = 6.21$, and $k_b^{imp} = 39.1$) and from clear-water conditions only $\varepsilon_{Q_s=0}$ (with $k_{b,Q_s=0}^{pack} = 6.60$, and $k_{b,Q_s=0}^{imp} = 29.0$).

	Sand mass feeding rate Q_s ($kg\ s^{-1}$)	MAE $\varepsilon_{Q_s=0}$ (kg)	MAE ε (kg)	MAE $\Delta\varepsilon$ (%)	RMSE $\varepsilon_{Q_s=0}$ (kg)	RMSE ε (kg)	RMSE $\Delta\varepsilon$ (%)	MAPE $\varepsilon_{Q_s=0}$ (%)	MAPE ε (%)	MAPE $\Delta\varepsilon$ (%)
Packets	0.0	0.272	0.259	-4.71	0.410	0.395	-3.67	27.9	30.4	+9.08
	0.90	0.283	0.256	-9.52	0.451	0.401	-11.0	26.8	28.8	+7.26
	1.35	0.296	0.267	-9.90	0.499	0.442	-11.4	22.8	23.9	+4.83
	1.80	0.289	0.254	-12.3	0.458	0.405	-11.5	28.7	28.4	-0.94
Impulses	0.0	0.184	0.421	+128	0.279	0.646	+131	29.4	40.5	+37.7
	0.90	0.945	0.279	-70.5	1.818	0.430	-76.4	54.6	29.7	-45.5
	1.35	0.987	0.208	-79.0	1.848	0.367	-80.2	52.5	23.2	-55.8
	1.80	1.106	0.254	-77.1	2.069	0.457	-77.9	61.6	25.5	-58.7

Table S6. Comparison of error metrics for predicted mass transport rates based on a power law relationship, derived from all experiments feeding rate ε and from clear-water conditions only $\varepsilon_{Q_s=0}$.
